# Preparation, Characterization and Properties of Porous PLA/PEG/Curcumin Composite Nanofibers for Antibacterial Application

**DOI:** 10.3390/nano9040508

**Published:** 2019-04-02

**Authors:** Feifei Wang, Zhaoyang Sun, Jing Yin, Lan Xu

**Affiliations:** National Engineering Laboratory for Modern Silk, College of Textile and Engineering, Soochow University, 199 Ren-ai Road, Suzhou 215123, China; ffwang@stu.suda.edu.cn (F.W.); zhaoyangsun1991@163.com (Z.S.); 20184215040@stu.suda.edu.cn (J.Y.)

**Keywords:** electrospinning, curcumin, PLA/PEG/curcumin nanofiber, drug release, porous nanofiber

## Abstract

Polylactide/polyethylene glycol/curcumin (PLA/PEG/Cur) composite nanofibers (CNFs) with varying ratios of PEG were successfully fabricated by electrospinning. Characterizations of the samples, such as the porous structure, crystalline structure, pore size, wetting property and Cur release property were investigated by a combination of scanning electron microscopy (SEM), Fourier-transform infrared (FTIR) spectroscopy, X-ray diffraction (XRD) and UV spectrophotometer. The antibacterial properties of the prepared porous CNFs against *Escherichia coli* bacteria were studied. The results showed that with the decrease of PEG in the CNFs, there appeared an evident porous structure on the CNF surface, and the porous structure could enhance the release properties of Cur from the CNFs. When the weight ratio (PEG:PLA) was 1:9, the pore structure of the nanofiber surface became most evident and the amount of Cur released was highest. However, the antibacterial effect of nonporous CNFs was better due to burst release over a short period of time. That meant that the porous structure of the CNFs could reduce the burst release and provide better control over the drug release.

## 1. Introduction

Electrospinning has been recognized as a simple and efficient technique for the fabrication of composite nanofibers (CNFs). The application of electrospun nanofibers can be improved by increasing the specific surface area and porosity of the nanofibers. Because of high porosity, high specific surface area and high surface activity, porous nanofibers have many existing and potential applications in drug delivery, tissue engineering, electronic engineering, and so on [1,2,3,4,5].

Curcumin (Cur) is a polyphenol compound extracted from the rhizome of the plant *Curcuma longa*. Recently, it was reported that Cur has a wide range of pharmacologic activities, such as anti-inflammation, anti-human immunodeficiency virus (HIV), anti-microbial, anti-oxidant, anti-parasitic, anti-mutagenic and anti-cancer, with low or no intrinsic toxicity [6,7,8]. Polylactic acid (PLA) has been widely used in biomedical and tissue engineering because of its excellent biocompatibility and biodegradability [9]. Lu et al. [10] investigated the effects of electrospraying a solution system and concentration of polyethylene oxide (PEO) on the morphologies of the single-drug and dual-drug loaded nanocomposites. Zhang et al. [11] reported that the polyethylene glycolation (PEGylation) modification could afford a faster release profile of the encapsulated drug than pure poly(lactic-co-glycolic acid) (PLGA) nanofibers, and the drug-loaded PLGA-PEG nanofibers were able to inhibit the growth of a model bacterium, *Staphylococcus aureus*. Ramírez-Agudelo et al. [12] found that composite nanofibers were easier to obtain an antibacterial property compared to a single polymer system. And the antibacterial composite with bacterial cellulose could prolong the antimicrobial activity against both *E. coli* and *S. aureus* [13]. Phan et al. [14] prepared and characterized a series of polymeric micellar formulations of Cur for targeted cancer therapy. Aytac et al. [15] designed core-shell nanofibers via coaxial-electrospinning using an inclusion complex of curcumin with cyclodextrin in the core and polylactide (PLA) in the shell. Zhong et al. [16] prepared PLA and a PLA-b-PEG composite porous scaffold loaded with a high dose of aspirin, using the solvent casting/particulate leaching technique, and found that the amphiphilic block polymer could efficiently enhance the dispersion property and stabilize the release of hydrophilic drugs. Shaik et al. [17] investigated the preventive role of orally administered *Aloe vera* supplemented probiotic *lassi* (APL) on *Shigella dysenteriae* infection in mice, and found that the immunoprotective effects of APL against *Shigella dysenteriae* induced infection in mice. Feng et al. [18] used tea polyphenol (TP) as an additive to develop to inhibit the spoilage of fish fillet during cold storage.

In this paper, electrospun PLA/PEG/Cur porous CNFs were fabricated by controlling the weight ratio of PEG:PLA. The effects of this ratio on the morphology and porous structure of CNFs were studied by scanning electron microscopy (SEM) and capillary flow porometry. Successful entrapments of Cur in the PEG/PLA CNFs were validated by Fourier-transform infrared (FTIR) spectroscopy and X-ray diffraction (XRD). Then, the properties of these CNFs with various PEG:PLA ratios were investigated by a contact angle (CA) measurement apparatus, UV spectrophotometer and shaking incubator. The results showed the CA and the cumulative release of Cur increased with the decrease of PEG, due to the appearance of a porous structure on the nanofiber surface. However, the antibacterial effect of the nonporous composite nanofiber membranes (CNFMs) was better due to burst release over a short period of time. The initial burst release of Cur was prevented by the porous structure of the porous CNFs compared to nonporous CNFs. That meant that the porous structure of CNFs could reduce the burst release and allow better control of the drug release.

## 2. Experimental

### 2.1. Materials

Polylactic acid (PLA), with an average molecular weight of 22,000 g/mol, was purchased from Anqing chemical Co. Ltd. (Anhui, China). The polyethylene glycol (PEG) with an average molecular weight of 400 g/mol was supplied by Sangon Biotech Co. Ltd. (Shanghai, China). The *N*,*N*-Dimethylformamide (DMF) (Analytical Reagent), chloroform (CF) (Analytical Reagent) and phosphate buffered saline (PBS) (Analytical Reagent, pH 7.4) were purchased from Shanghai Chemical Reagent Co. Ltd. (Shanghai, China). The curcumin (Cur) (≥95.0% purity) was purchased from Shanghai Yuanye Biotechnology Co. Ltd. (Shanghai, China). *E. coli* for antibacterial tests were obtained from Soochow University (Suzhou, China). The nutrient broth medium and nutrient agar medium were purchased from SCAS Ecoscience Technology Inc. (Shanghai, China). All chemicals were used without further purification.

### 2.2. Preparation of PLA/PEG/Cur CNFs

The concentration was measured by weight. PEG and PLA with various weight ratios of 1:1, 1:3, 1:5, 1:7 and 1:9 were respectively dissolved in a solvent mixture of DMF and CF, with a weight ratio of 1:9. The concentrations of mixed solutions (PEG and PLA weight ratio to solution) were all 8 wt%. Then, a certain amount of Cur was added into the PEG/PLA solutions and the weight ratio of Cur to PEG/PLA was 3:100. The prepared solutions were magnetically stirred for 3 h to ensure homogeneous mixing.

The obtained spinning solution was dropped into a 10 mL syringe. According to our previous work [1,5], the electrospinning parameters were set as follows: The flow rate was 0.6 mL/h, the applied voltage was 15 kV, and the working distance from the needle tip to the collector was 14 cm. All electrospinning experiments were carried out at room temperature (20 °C) and a humidity of 60%.

### 2.3. Characterizations and Measurements

The electrical conductivity and viscosity of spinning solutions were determined respectively by a conductivity meter (DDS-307, Shanghai instrument & electric Scientific Instrument Co., Ltd., Shanghai, China) and a viscometer (NDJ-5S, Shanghai Nirun Intelligent Technology Co., Ltd., Shanghai, China). The measurement was repeated three times.

The surface morphologies of PLA/PEG/Cur CNFs were carried out using a scanning electron microscopy (SEM, Hitachi S-4800, Tokyo, Japan) at an acceleration voltage of 3 KV. The diameter distribution of the nanofibers was characterized by Image J software (National Institute of Mental Health, Bethesda, MD, USA). Twenty SEM images of 50 nanofibers in each SEM image were chosen at random for diameter distribution analysis.

The mixture, consisting of 1 mg of shredded PLA/PEG/Cur CNFMs and 200 mg of KBr powder, was pressed into a flaky sample, which was used in the Fourier-transform infrared (FTIR) analysis. The sample was characterized using FTIR spectroscopy (Nicolet 5700, Thermo Fisher Scientific, Waltham, WA, USA). The FTIR spectrum of the sample was obtained by the performance of 32 scans with the wavenumber range of 400–4000 cm^−1^ and a resolution of 4 cm^−1^.

X-ray diffraction (XRD) analyses were performed to illustrate the crystalline structures of Cur powders and PLA/PEG/Cur CNFs using a Philips X’Pert-Pro MPD with a 3 KW ceramic tube as the X-ray source (Cu-Kα) and an X’Celerator detector. Cu-Kα radiation was used with a diffraction angle range of 10–60° at 40 kV and 200 mA at a scanning rate of 10°/min.

The pore size distributions of PLA/PEG/Cur CNFMs were determined using capillary flow porometry (Porometer 3G, Quantachrome Instruments, Boynton, FL, USA), which employed the technique of expelling a wetting liquid, Porofil, from through-pores in the sample. All samples were circular membranes with a diameter of 25 mm and a thickness of 10 μm.

The wettability of the PLA/PEG/Cur CNFMs was characterized using an optical contact angle (CA) measurement instrument (Krüss DSA100, Krüss Company, Hamburg, Germany). The volume of the deionized water droplet used for static CA measurement was 6 μL. The CAs were measured by the sessile drop method [19]. Moreover, the average CAs were determined by measuring six different positions of the same sample.

### 2.4. In Vitro Release of Cur

Electrospun PLA/PEG/Cur CNFs (10 mg) were dispersed in 3 mL of PBS (PH 7.4) as a release medium and then placed in 25 mL centrifuge tubes (Shanghai Hongsheng Biotech Co. Ltd., Shanghai, China). These centrifuge tubes were incubated in a shaking incubator (FLY-100/200, Shanghai Shenxian Thermostatic Equipment Factory, Shanghai, China) at 37.4 °C with a shaking speed of 60 rpm. At desired intervals, 1 mL of the release medium was withdrawn for Cur release rate analysis, and an equal volume of fresh PBS was replenished for sustaining the incubation. The released amounts of Cur were measured by UV spectrophotometer (Cary 5000, Agilent Technologies, Santa Clara, CA, USA).

### 2.5. Antimicrobial Tests

The PLA/PEG/Cur CNFMs were cut into 5 mm × 5 mm shreds and were sterilized for 30 min, at a temperature of 125 °C and a pressure of 10^3^ KPa. The antibacterial effects of these PLA/PEG/Cur CNFMs on a bacterial strain, *E. coli*, were investigated. Overnight cultures of *E. coli* derived from a single colony and cultivated in nutrient broth medium were used in the study. Each culture solution (1 mL) was inoculated into 9 mL of PBS, resulting in a concentration of 3 × 10^5^–5 × 10^5^ colony forming units (CFUs/mL). These bacterial solutions with a concentration of 3 × 10^5^–5 × 10^5^ CFUs/mL were used for the antibacterial tests, and bacterial solutions incubated in pure PBS only served as controls.

In a conical flask, 375 mg of shredded PLA/PEG/Cur CNFMs were dispersed in 35 mL of PBS, and then 5 mL of bacterial solutions with a concentration of prepared 3 × 10^5^–5 × 10^5^ CFUs/mL were added. The conical flask was shaken in a constant temperature incubator shaker (IS-RDV1, Crystal Technology & Industries, Inc., Dallas, TX, USA) at 25 °C with a shaking speed of 300 rpm for 20 h. Aliquots (1 mL) of the obtained samples were placed on nutrient agar plates in multiple replicates. Each sample was prepared separately in a triplicate. The growth of the *E. coli* was evaluated by counting CFUs after incubation for 24 h at 37 °C. The inhibition rate was calculated according to the following equation [20]:

$$\mathrm{Inhibition}\;\mathrm{rate}\;(\%)\;=\frac{\mathrm{CFUcontrol}\;\mathrm{group}\;-\;\mathrm{CFUexperimental}\;\mathrm{group}\;}{\mathrm{CFUcontrol}\;\mathrm{group}}\times 100\%$$

## 3. Results and Discussion

### 3.1. Properties Characterization of Spinning Solutions

The electrical conductivity and viscosity of the spinning solutions with the different weight ratios (PEG:PLA) are shown in Table 1. It was obvious that as the content of the PLA increased, the viscosity of solutions increased due to increased average molecular weight [21], which resulted in a decrease of the electrical conductivity of the solutions [22]. The viscosity played an important role in determining the diameter of the electrospun nanofibers [1].

### 3.2. Morphological Characterization of PLA/PEG/Cur CNFs (SEM)

**Figure 1:**
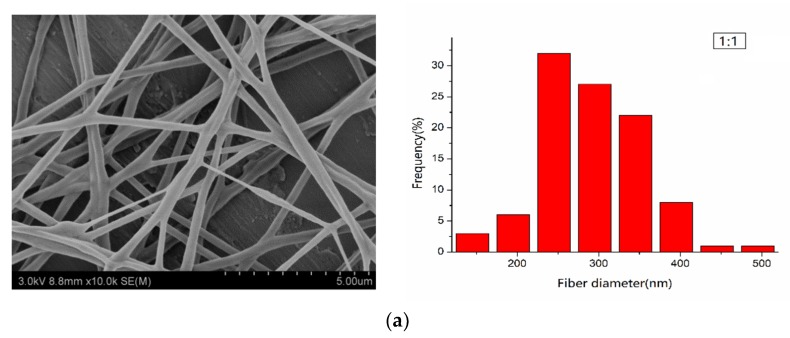

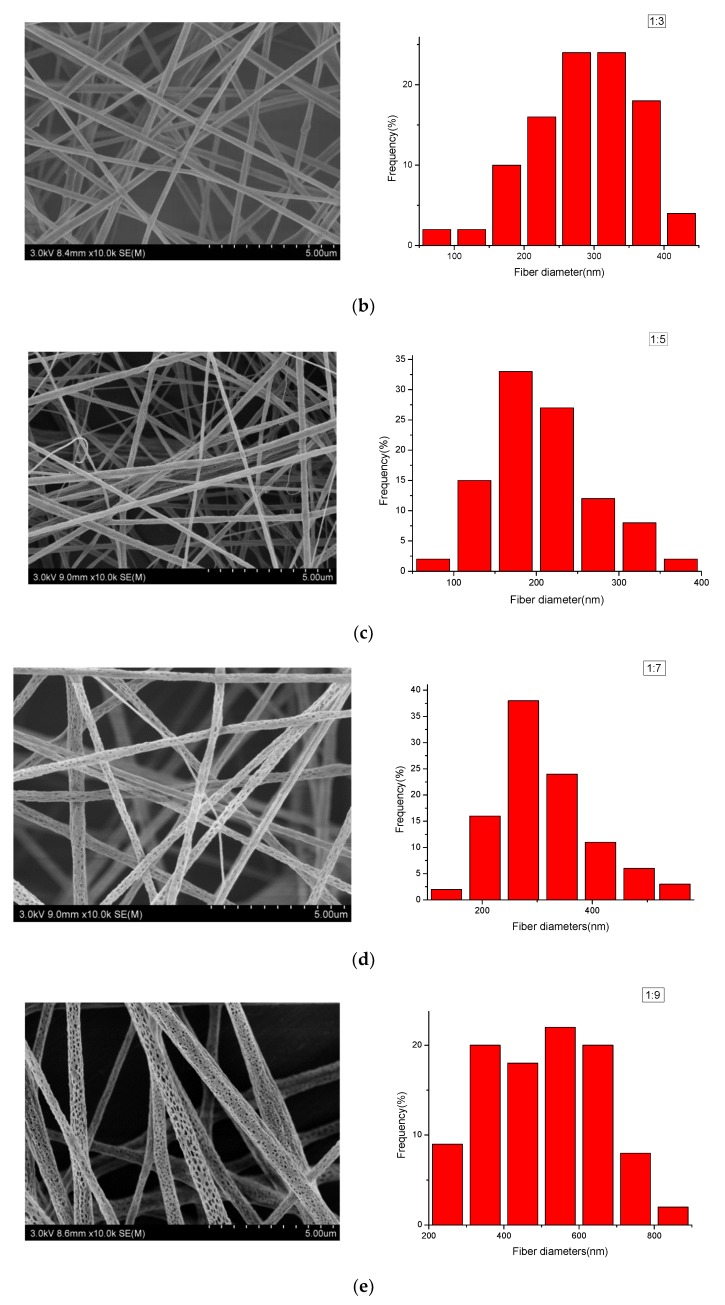
SEM pictures of the electrospun polylactide/polyethylene glycol/curcumin (PLA/PEG/Cur) carbon nanofibers (CNFs) with different weight ratios (PEG:PLA). The right figures were the according diameter distribution: (**a**) 1:1, (**b**) 1:3, (**c**) 1:5, (**d**) 1:7, (**e**) 1:9.

The surface morphologies of the electrospun PLA/PEG/Cur CNFs with different weight ratios (PEG:PLA) were investigated by SEM. Figure 1 shows SEM images of the CNFs and the respective nanofiber diameter distributions. It could be seen that with the decrease in the weight ratio (PEG:PLA), the surface morphology of the CNFs changed from smooth to porous. A further decrease of PEG in the mixtures, with the weight ratio 1:5, resulted in the appearance of evident pores on the CNF surfaces. When the weight ratio (PEG:PLA) was 1:7 and 1:9, the pore structure of the nanofiber surfaces became very noticeable, and the diameters of the porous nanofibers increased due to an increased pore number. This could be explained by the plastic deformation of PLA in water, which led to the formation of the pore structure, due to the solvent volatilization at a suitable humidity in the electrospinning process. Therefore, as the PLA content increased to a certain proportion, evident pore structures appeared on the nanofiber surfaces.

In addition, the relationship between the weight ratio (PEG:PLA) and the average diameters of the electrospun CNFs is shown in Table 2, with the confidence intervals calculated according to Reference [1]. Table 2 illustrates that with the decrease of the weight ratio (PEG:PLA), the average diameters of the CNFs decreased. This was firstly due to better spinnability of the spinning solution, which increased rapidly with a weight ratio of 1:7, due to the formation of pores [21]. When the contents of PEG were too much, such as 1:1 and 1:3 (mass ratios to PLA), the viscosity of the spinning solution was too low, which resulted in poor spinnability of the solution.

### 3.3. Fourier-Transform Infrared (FTIR) and Raman Spectrum Analysis

**Figure 2:**
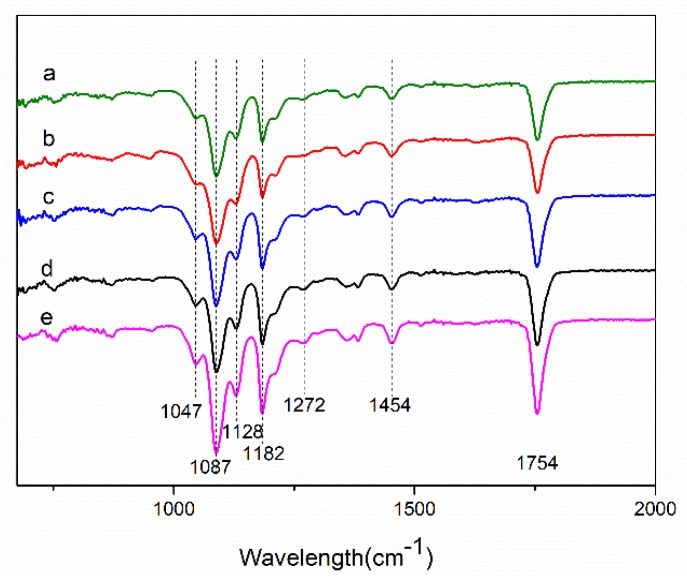
Fourier-transform infrared (FTIR) spectra of PLA/PEG/Cur CNFs with different weight ratios (PEG:PLA): (**a**) 1:1; (**b**) 1:3; (**c**) 1:5; (**d**) 1:7; (**e**) 1:9.

The presences of Cur in the PEG/PLA CNFs with the different weight ratios (PEG:PLA) were confirmed by Fourier-transform infrared (FTIR) spectroscopy, as shown in Figure 2. The FTIR spectra of these PLA/PEG/Cur CNFs displayed characteristic absorption bands at 1754 cm^−1^ and 1087 cm^−1^, which represented the backbone ester group of PLA [4], and also showed a CH_3_ asymmetric bending peak at 1454 cm^−1^, a C-O stretching peak at 1182 cm^−1^ and a O-H bending peak at 1047 cm^−1^ of PLA. In addition, the absorption peak at 1128 cm^−1^ could be attributed to C-O-C characteristic vibration of PEG [23]. Moreover, it was obvious that a sharp peak appeared at 1272 cm^−1^ due to the C-O stretching vibration in -C-OCH_3_ of the phenyl ring of Cur [24]. As seen in Figure 2, Cur was encapsulated in the CNFs, and the intensity of the peaks corresponding to PLA in FTIR spectra of samples increased as the PLA contents increased.

### 3.4. X-ray Diffraction (XRD) Spectrum Analysis

**Figure 3:**
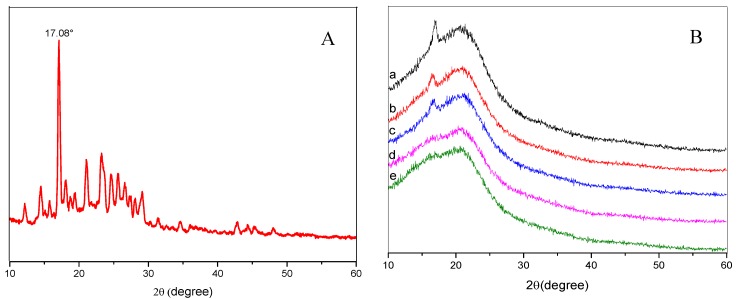
XRD spectra of Cur (**A**). XRD spectra of PLA/PEG/Cur CNFs with the different weight ratio (PEG:PLA): (**a**) 1:1; (**b**) 1:3; (**c**) 1:5; (**d**) 1:7; (**e**) 1:9 (**B**).

XRD patterns with distinctive crystalline peaks of Cur and PLA/PEG/Cur CNFs with different weight ratios (PEG:PLA) are shown in Figure 3. As seen in Figure 3A, the XRD spectrum of Cur displayed sharp and intense peaks of crystallinity, which suggested a highly crystalline nature. Figure 3B shows the XRD spectra of the CNFs with different weight ratios (PEG:PLA). It could be observed that the peaks at 2θ = 16.88° and 22.58° could be assigned as (010) crystal planes of PEG and (015) crystal planes of PLA, respectively. With the decrease of PEG, the XRD spectra of the CNFs showed a reduction of peak intensity, as compared to the Cur, which indicated decreased crystallinity or changes into an amorphous phase of the drug [6]. The principal peak of Cur at 2θ = 17.08° did not appear due to the low ratio of Cur with respect to the polymer composite.

### 3.5. Measuring Pore Size Distribution Composite Membranes

**Figure 4:**
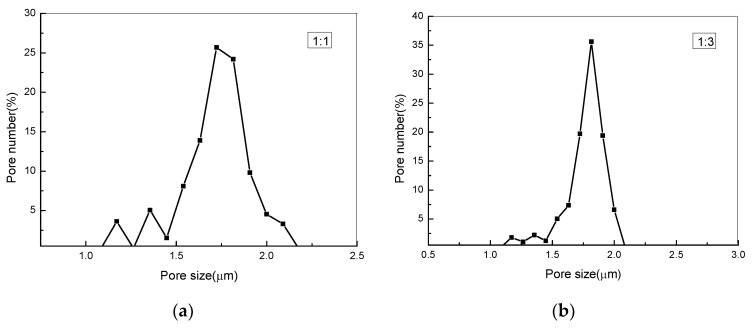

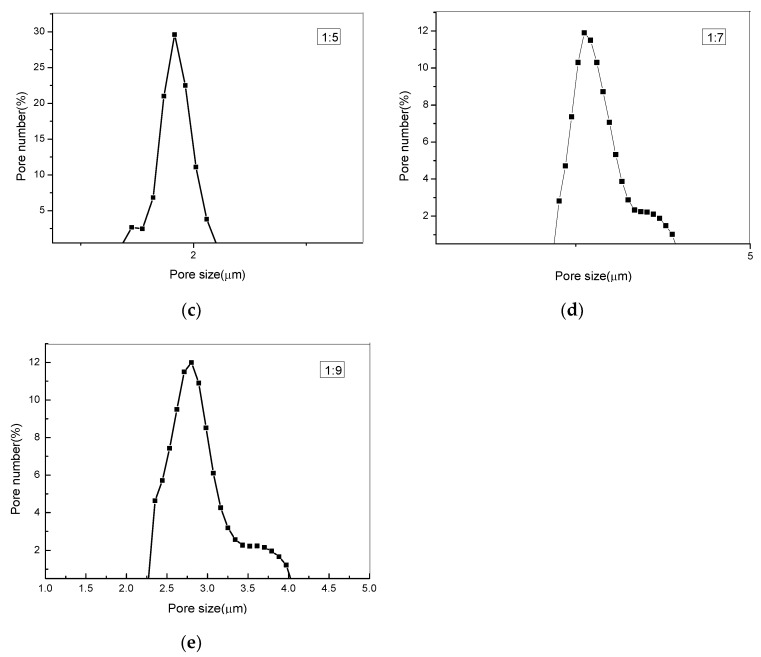
The pore size distributions of PLA/PEG/Cur nonporous composite nanofiber membranes (CNFMs) with different weight ratios (PEG:PLA): (**a**) 1:1; (**b**) 1:3; (**c**) 1:5; (**d**) 1:7; (**e**) 1:9.

Figure 4 illustrates the pore size distributions of the PLA/PEG/Cur CNFMs with varying PEG weight ratios, measured by a capillary flow porometry, and Table 3 shows the size and number of pores. It could be seen that as the PEG weight ratio decreased, the pore size of the CNFMs increased, and the respective pore number of the CNFMs decreased. Moreover, the pore distributions of the CNFMs with the weight ratios 1:7 and 1:9 (PEG:PLA) demonstrated more uniformity. These results were in agreement with the SEM images, as illustrated in Figure 1.

### 3.6. Wetting Properties

The CA values of the PLA/PEG/Cur CNFMs with different weight ratios (PEG:PLA) were obtained by an optical (CA) measurement instrument (Table 4). It was obvious that the CNFMs with the weight ratio 1:1 (PEG:PLA) were hydrophilic due to the hydrophilicity of PEG, and with the increase of the PLA weight ratio, the CA increased gradually due to the hydrophobicity of PLA. When the weight ratio was up to 1:3, the CA of the CNFMs increased to 125.4°. With the continuous increase of the PLA weight ratio, the CNFMs were highly hydrophilic. This might be due to the increase of hydrophobic PLA and pores of the CNFs. The pore structure could further enlarge the surface area and enhance the hydrophobicity of the CNFMs [25].

### 3.7. In Vitro Cur Release

**Figure 5:**
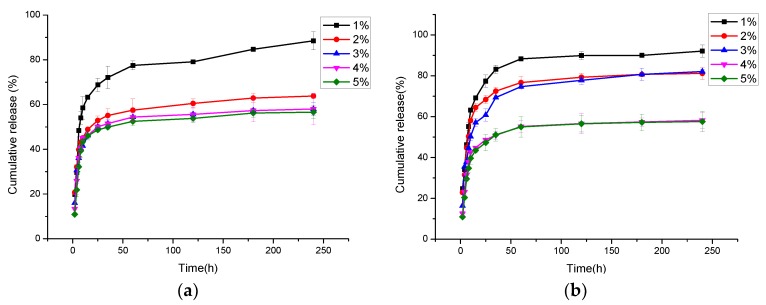
Cumulative release of Cur from porous and nonporous PLA/PEG/Cur CNFs with the different Cur contents: (**a**) Nonporous CNFs; (**b**) Porous CNFs.

The released amounts of Cur in PBS from the electrospun CNFs were determined spectrophotometrically at 425 nm by a UV spectrophotometer. The standard curve of Cur concentration was calculated according to *y* = 14.26087*x* − 0.06634 (R = 0.997), where *x* is the Cur concentration (µg/mL), *y* is the optical density (OD) value measured. Based on the standard curve, the cumulative release of Cur was calculated. Figure 5 illustrates the cumulative release of Cur from the porous and nonporous CNFs with the different Cur contents, which were prepared with a weight ratio of 1:7 (PEG:PLA), at a humidity of 60% (porous) and 30% (nonporous), respectively. It could be seen that after 240 h the cumulative release rates of Cur from the porous CNFs with varying Cur percentages from 1% to 5%, were 92.17%, 81.23%, 82.89%, 58.36%, and 57.62%, respectively, were higher than those from the according nonporous CNFs, which were 88.51%, 63.83%, 58.10%, 58.35% and 56.66%. This may have been due to the higher specific surface area of the porous nanofibers and the porous structure could promote the release of the drug [4]. In addition, the cumulative release rate of Cur from the CNFs with 1% Cur was highest, and when the Cur percentage was higher than 3%, the cumulative release rate of Cur was at a low level. This may have been due to the fact that Cur could be dissolved less in the spinning solution when the Cur percentage was at a higher level. Therefore, 3% Cur was selected as the experimental parameter in the following study.

**Figure 6:**
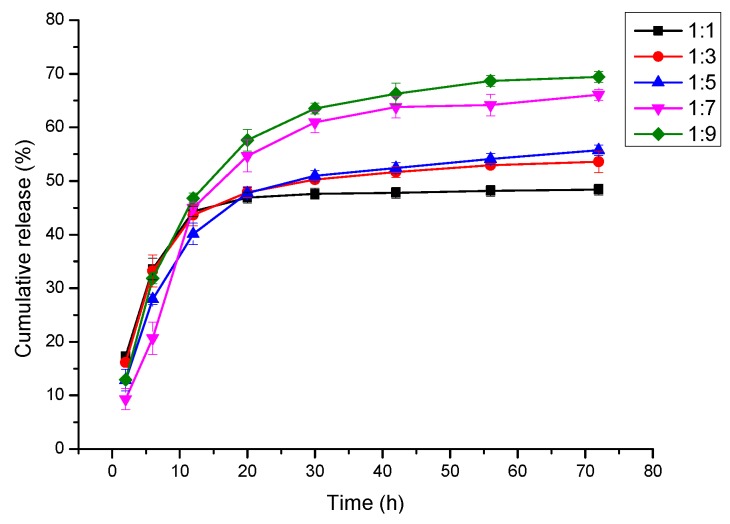
Cumulative release of Cur from PLA/PEG/Cur CNFs with the different weight ratio (PEG:PLA).

Figure 6 shows the cumulative release of Cur from the PLA/PEG/Cur CNFs with 3% Cur and different weight ratios (PEG:PLA). It was evident that after 80 h, the Cur release of CNFs with different weight ratios (PEG:PLA), such as 1:1, 1:3, 1:5, 1:7 and 1:9, reached 48.40%, 53.58%, 55.75%, 66.08% and 69.41%, respectively. That meant the Cur release increased as the PLA weight ratio increased, due to the appearance and increase of pores on the CNFs. The results also demonstrated that the porous structure of the CNFs could absorb more Cur and promote the release of Cur. Therefore, the PLA/PEG/Cur porous CNFs exhibited an improved drug release property.

### 3.8. Effects of Porous Structure on Antibacterial Properties

**Figure 7:**
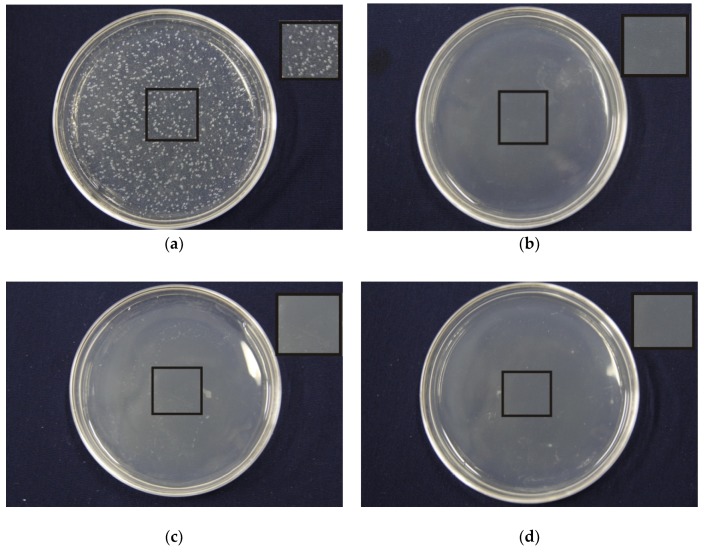

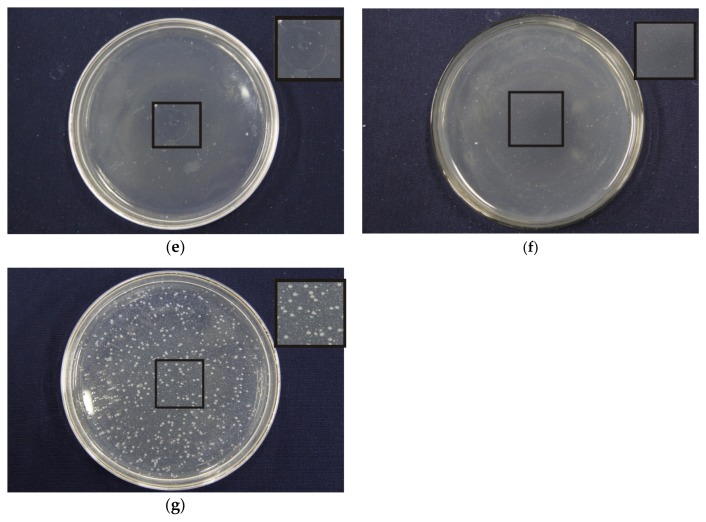
Effects of different composite ratio on antibacterial properties of PLA/PEG/Cur nanofibers membranes: (**a**) PEG:PLA = 1:7 (0% Cur); (**b**) PEG:PLA = 1:1 (3% Cur); (**c**) PEG:PLA = 1:3 (3% Cur); (**d**) PEG:PLA = 1:5 (3% Cur); (**e**) PEG:PLA = 1:7 (3% Cur); (**f**) PEG/PLA = 1:9 (3% Cur); (**g**) cotton.

**Figure 8:**
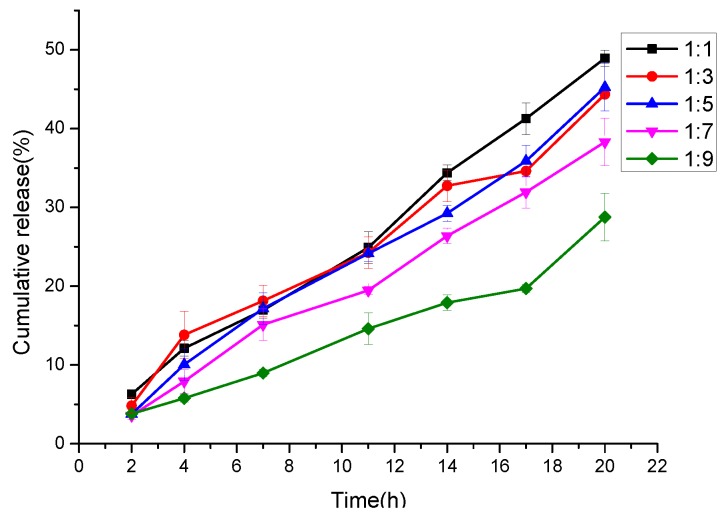
Initial burst release of Cur from PLA/PEG/Cur CNFMs with different weight ratios (PEG:PLA).

Figure 7 and Table 5 present the antibacterial properties of the PLA/PEG/Cur CNFMs with 3% Cur and different weight ratios (PEG:PLA), and the PEG/PLA CNFMs with a weight ratio (PEG:PLA) of 1:7 used as a comparative sample. It could be seen that the antibacterial effect of the PEG/PLA CNFMs with a weight ratio of 1:7 was poor, and the inhibition rates of the PLA/PEG/Cur CNFMs were all beyond 97%. The antibacterial effect of the PLA/PEG/Cur CNFMs was as good as that of other antibacterial materials in Reference [26]. However, the inhibition rates decreased as the PLA weight ratio increased. To illustrate the results of the antibacterial tests, the initial burst releases of Cur from the PLA/PEG/Cur CNFMs with 3% Cur and different weight ratios (PEG:PLA) for about 20 h were investigated. The standard curve of the Cur concentration was calculated according to *y* = 186.428*x* − 3.3719 (R = 0.997). According to the standard curve, the cumulative releases of Cur were calculated, as displayed in Figure 8.

As seen in Figure 8, as the PLA weight ratio increased, the initial burst release rate of Cur decreased due to the appearance and increase of pores on the CNFs, which resulted in the decrease of the inhibition rates of the CNFMs. The results showed that the antibacterial effect of the nonporous CNFs was better due to burst release over a short period of time. This meant that the porous structure of the CNFs could reduce the initial burst release and better control drug release. This could be explained using a modified Fickian diffusion equation, which is presented in our previous work [4]. According to the equation, the porous nanofibers had a longer diffusion path than the nonporous nanofibers, and as the diffusion path increased, the concentration gradient of the drug would become smaller. As a result, the diffusion flux would decrease and lead to the slow release of the drug [4].

## 4. Conclusions

In the present study, electrospun PLA/PEG/Cur porous CNFs with different weight ratios (PEG:PLA) were successfully prepared by controlling the electrospinning parameters. The electrical conductivity and viscosity of the spinning solutions with different weight ratios (PEG:PLA) were investigated. The results indicated that with the decrease in the weight ratio, the electrical conductivity of spinning solutions decreased, and accordingly, the viscosity of the solutions increased.

The effects of the weight ratio (PEG:PLA) on the morphology and structure of the CNFs were studied systematically. SEM images displayed that as the weight ratio decreased, the morphology of the CNFs changed from smooth to porous. And when the weight ratio increased to 1:7, the pore structure of the nanofiber surfaces became very evident. FTIR and XRD data indicated that Cur was encapsulated successfully in the CNFs. Pore size analysis displayed that with the decrease of the weight ratio, the pore size of the CNFMs increased, and the respective pore number of the CNFMs decreased. CA measurements illustrated that the CA of the CNFMs increased as the weight ratio decreased.

Property characterizations—such as the wetting property, drug release property and antibacterial property—of the PLA/PEG/Cur CNFMs showed that with the decrease of the weight ratio, the CA and the Cur cumulative release of the CNFMs increased due to the appearance of pores on the CNFs. In addition, the antibacterial effect of the CNFMs decreased, as the initial burst release of Cur was prevented by the porous structure of the CNFs. Therefore, the porous structure of the CNFs could improve the drug release property, and the PLA/PEG/Cur porous CNFMs could have great potential for biomedical applications, such as drug delivery, biological scaffold, medical dressing and antibacterial materials.

## Figures and Tables

**Table 1:** Properties characterization of spinning solutions with the different weight ratio (polyethylene glycol:polylactide, or PEG:PLA).

PEG:PLA	Electrical Conductivity (μs/cm)	Viscosity (mPa·s)
1:1	0.455 ± 0.045	33 ± 2
1:3	0.432 ± 0.038	77 ± 4
1:5	0.421 ± 0.035	112 ± 3
1:7	0.267 ± 0.052	137 ± 3
1:9	0.249 ± 0.048	157 ± 4

**Table 2:** The relationship between the weight ratio (PEG:PLA) and the average diameters of CNFs.

PEG:PLA	Average Diameter $\left(\bar{D}\right)$ (nm)	Standard Deviation (*σ*) (nm)	Confidence Interval (nm)
1:1	290.60	64.08	±12.56
1:3	287.70	74.39	±16.91
1:5	205.16	59.43	±11.64
1:7	308.73	91.25	±17.89
1:9	552.81	195.53	±38.32

**Table 3:** The analysis of pore size distributions of the CNFMs with different weight ratios (PEG:PLA).

PEG:PLA	Pore Size (μm)	Maximum Pore Number (/cm^2^) /The According Pore Size (μm)	Pore Number (/cm^2^)
1:1	1.39–2.11	4.3 × 10^7^/1.769	1.68 × 10^8^
1:3	1.41–2.18	4.13 × 10^7^/1.861	1.15 × 10^8^
1:5	1.55–2.39	2.2 × 10^7^/1.879	7.4 × 10^7^
1:7	2.35–3.93	5.6 × 10^6^/2.668	5.1 × 10^7^
1:9	2.44–4.04	5.5 × 10^6^/2.848	4.5 × 10^7^

**Table 4:** Contact angles of PLA/PEG/Cur CNFMs with different weight ratios (PEG:PLA).

Ratio	1:1	1:3	1:5	1:7	1:9
**Contact angles**	47.1 ± 1.1°	125.4 ± 2.6°	130.2 ± 1.1°	134.6 ± 2.0°	139.0 ± 1.7°

**Table 5:** Effects of different composite ratio on antibacterial properties of PLA/PEG/Cur nanofiber membranes.

PEG/PLA	Inhibition Rates (%)
1:1	99.97
1:3	99.96
1:5	99.93
1:7	98.77
1:9	97.61
